# Supplementary material for: P-TRAP: a Panicle Trait Phenotyping tool
Source: BMC Plant Biol. 2013 Aug 29;13:122. doi: 10.1186/1471-2229-13-122 (PMC3848748; doi:10.1186/1471-2229-13-122)
Supplement: Additional file 7 — P-TRAP source code. The Netbeans project that contains the source code of the application. [file 1471-2229-13-122-S7.zip › javadoc.zip!/javadoc/org-biolag-particleprocessor/org/biolag/particleprocessor/ParticlesModel.html]

ParticlesModel (ParticleProcessor)


JavaScript is disabled on your browser.


- Package
- Class
- Tree
- Deprecated
- Index
- Help

*org.biolag.particleprocessor 1.0*

- Prev Class
- Next Class

- Frames
- No Frames

- All Classes

- Summary:
- Nested |
- Field |
- Constr |
- Method

- Detail:
- Field |
- Constr |
- Method


org.biolag.particleprocessor

## Class ParticlesModel

- java.lang.Object
- - org.biolag.particleprocessor.ParticlesModel

- ---

    

  ```
  public class ParticlesModel
  extends java.lang.Object
  ```

  A model class for I/O manipulating of the particles.

  Author:
  :   faroq

- - ### Constructor Summary

    Constructors

    Constructor and Description

    `ParticlesModel()`

    `ParticlesModel(java.util.ArrayList<Particle> particles)`

    `ParticlesModel(org.w3c.dom.Element particles)`

    Create a ParticlesModel from an XML file.
  - ### Method Summary

    Methods

    | Modifier and Type | Method and Description |
    | --- | --- |
    | `void` | `calcAll()` Calculate all traits. |
    | `double` | `getAR()` |
    | `double` | `getarea()` |
    | `double` | `getcircularity()` |
    | `double` | `getcompactness()` |
    | `double` | `getellipciticiy()` |
    | `double` | `getheightAverage()` |
    | `java.util.ArrayList<Particle>` | `getparticles()` |
    | `double` | `getprimeter()` |
    | `int` | `getProportionalitySum()` The number of particles. |
    | `double` | `getwidthAverage()` |
    | `int` | `size()` |
    | `java.lang.String` | `toString()` |

    - ### Methods inherited from class java.lang.Object

      `clone, equals, finalize, getClass, hashCode, notify, notifyAll, wait, wait, wait`

- - ### Constructor Detail


    - #### ParticlesModel

      ```
      public ParticlesModel(java.util.ArrayList<Particle> particles)
      ```


    - #### ParticlesModel

      ```
      public ParticlesModel()
      ```


    - #### ParticlesModel

      ```
      public ParticlesModel(org.w3c.dom.Element particles)
      ```

      Create a ParticlesModel from an XML file.

      Parameters:
      :   `particles` - and XML element
  - ### Method Detail


    - #### size

      ```
      public int size()
      ```


    - #### getparticles

      ```
      public java.util.ArrayList<Particle> getparticles()
      ```


    - #### toString

      ```
      public java.lang.String toString()
      ```

      **Overrides:**
      :   `toString` in class `java.lang.Object`


    - #### getProportionalitySum

      ```
      public int getProportionalitySum()
      ```

      The number of particles.

      Returns:


    - #### getwidthAverage

      ```
      public double getwidthAverage()
      ```


    - #### getheightAverage

      ```
      public double getheightAverage()
      ```


    - #### getarea

      ```
      public double getarea()
      ```


    - #### getprimeter

      ```
      public double getprimeter()
      ```


    - #### getcircularity

      ```
      public double getcircularity()
      ```


    - #### getellipciticiy

      ```
      public double getellipciticiy()
      ```


    - #### getcompactness

      ```
      public double getcompactness()
      ```


    - #### getAR

      ```
      public double getAR()
      ```


    - #### calcAll

      ```
      public void calcAll()
      ```

      Calculate all traits.


- Package
- Class
- Tree
- Deprecated
- Index
- Help

*org.biolag.particleprocessor 1.0*

- Prev Class
- Next Class

- Frames
- No Frames

- All Classes

- Summary:
- Nested |
- Field |
- Constr |
- Method

- Detail:
- Field |
- Constr |
- Method
